# Use of interactional metadiscourse and identity construction in responses to negative online reviews of Chinese and British hotels

**DOI:** 10.1371/journal.pone.0316071

**Published:** 2024-12-27

**Authors:** Jingjing Huang, Wei Xiao

**Affiliations:** School of Foreign Languages, Hefei Normal University, Hefei, China; University of Malaya Faculty of Education, MALAYSIA

## Abstract

In the business context, effectively responding to negative reviews is critical for a hotel to maintain reputation and customer relations. To explore the linguistic devices employed in addressing guest complaints, a corpus-based study is conducted on the use of interactional metadiscourse and identity construction in responses to negative online reviews of Chinese and British Hotels. Drawing upon the statistical results of the usage of interactional metadiscourse and the analysis of discourse examples, this study delves into the frequency and similarities/differences in the employment of five subcategories of interactional metadiscourse across the respective corpora of 100 responses to negative reviews from hotels in Beijing and hotels in London. Furthermore, the study examines the characteristics and similarities/ differences of the identity construction of manager, communicator, doer and advisor with the use of interactional metadiscourse. The findings reveal that there are significant differences between the two sides in the use of self-mentions, boosters, hedges and positive attitude markers, while there is little difference in the use of engagement markers and negative attitude markers. The most constructed identity by both sides is the communicator, and the least is the adviser, with little difference. The identity of manager is significantly more prevalent in responses from hotels in Beijing, whereas hotel responders in London exhibit a notably greater tendency to construct the identity of doer. The similarities and variances of interactional metadiscourse use and identity construction indicate the two sides’ distinctive priorities in interactions with guests and different cultural values, which provide valuable insights for hotels on the effective use of metadiscourse to construct multiple identities, revealing that the strategically crafted responses play a pivotal role in shaping favorable images, fostering harmonious relationships with customers and promoting sustainable development of the hotels.

## 1. Introduction

With the advent of the internet era, an increasing number of hotels are cooperating with travel websites to provide hotel booking services for guests and receive feedback from them. Guests share their reviews on travel websites, and hotel management should proactively monitor online comments and manage their online reputation by providing timely and genuine responses to reviews [1]. The presence of hotels’ inappropriate responses to reviews may have a negative impact on guests’ purchasing intentions [2]. Negative reviews also have a negative impact on a hotel’s image [3]. Therefore, effectively responding to guests’ negative reviews is crucial for managing a hotel and embodying a pivotal marketing tool to enhance or repair its business reputation [4, 5].

Business responses to negative reviews fall within the scope of language utilization, and scholars have delved into this topic through various perspectives, such as examining the underlying motivations behind customer grievances and the discursive strategies employed by businesses to salvage their images, like issuing apologies, expressing gratitude and providing explanations [6]. Additionally, studies have analyzed the different rhetorical moves and countermeasure strategies deployed in business responses to customer complaints [7], discussed the service recovery strategy with various moves, such as rectification, explanation and apology [8], as well as explored identity construction and relationship management in responses to online negative reviews [9]. Recent research focuses on the use of CHV (conversational human voice) in business responses [10]. Creelman [11] examines how customer care agents employ specific linguistic elements, politeness strategies and rhetorical moves to operationalize a conversational communication style so as to support brand-customer relationships; Holmgreen [12] explores the realization of CHV through the use of message personalization, informal speech and invitational rhetoric in bank employees’ responses to users’ criticism.

In the business contexts, the communicative and politeness norms differ across different languages and cultures [13]. Researchers have conducted cross-cultural studies on responses to negative business reviews, like analyzing the rhetorical moves structuring the responses to negative reviews and communicative strategies employed in English, Dutch and Italian [14], comparing the differences in speech acts and discourse strategies utilized in hotel apologies responding to negative reviews between English and Japanese [15], examining how tourists, while sharing their travel experiences, adopt uniform linguistic norms and communicative practices or exhibit distinct discursive preferences in reviews written in Spanish, Italian and French [16], comparing the move structure and dimensions of CHV (personalization, informality and invitational rhetoric) in a corpus of response emails to telecom sector complaints, drawn from both British and Peninsular Spanish contexts [17]. Researchers have placed significant emphasis on responders’ management of interpersonal relationships and their adept use of pragmatic strategies, while also observing the pragmatic variations in responses to negative reviews across different cultures. However, there remains a scarcity of cross-cultural studies examining the correlation between the use of metadiscourse and identity construction in the business context.

The appropriate use of responses to negative reviews is crucial for hotels in restoring their tarnished image, reestablishing harmonious guest relationships and enhancing their market competitiveness. Research on CHV underscores the importance of adopting an engaging and natural style in organizational communication with the public [18], highlighting the role of appropriate discourse in image restoration and rapport enhancement [11, 17]. This aligns with studies on metadiscourse, which focus on responders’ linguistic devices in expressing emotion and building relationships with guests. However, metadiscourse research in business responses extends beyond linguistic strategies, exploring how responders employ metadiscourse to effectively direct readers’ attention, manage expectations, and bolster the persuasiveness and acceptability of their responses at a meta-level. Thus, the manifestation of metapragmatic awareness in responses to negative reviews is worthy of further investigation.

Drawing upon the communicator’s metapragmatic awareness, which involves “using language to reflect on and articulate one’s understanding of various ways of interacting and communicating with others” [19], the hotel responders utilize interactional metadiscourse to engage in virtual interaction with guests and orchestrate discourse planning and management. In addition, metadiscourse is used to dynamically construct identities to achieve specific communicative goals [20]. Therefore, it is highly important to study the use of interactional metadiscourse and the construction of identities in hotel responses to negative reviews. Based on the responses to negative hotel reviews on TripAdvisor.com, this study explores the characteristics, similarities and differences of interactional metadiscourse employed by Chinese and British hotel responders, elucidating how they dynamically construct identities for the purpose of fostering harmonious relationships with guests and cultivating favorable hotel images.

## 2. Literature review

Since Harris [21] introduced the concept of metadiscourse, scholars have embarked on exploring its implications and functions from diverse perspectives. Studies approaching metadiscourse in a narrow sense concentrate solely on its role in organizing text [22, 23], while studies of metadiscourse in a broad sense encompass all linguistic forms tasked with organizing, classifying, interpreting and evaluating discourse information, emphasizing the dual functions of metadiscourse, namely, discourse organization and interpersonal interaction [24–26]. It can be broadly conceptualized as a linguistic, rhetorical and pragmatic resource that signifies and mirrors the interplay between the information of the message, the sender and the receiver [27]. Therefore, metadiscourse serves to “guide, direct and inform” the reader regarding the writer’s proposition [28], which plays a crucial role in facilitating effective communication between the two sides.

Hyland [29] further promotes the study of the interpersonal meaning of metadiscourse, pointing out that metadiscourse is a pivotal aspect of effective communication between the writer and the reader, encompassing elements such as discourse organization, the writer’s way of aiding readers in understanding the flow and logic of the text, and the writer’s attitude towards the content of the discourse. He also introduces an interpersonal model of metadiscourse, dividing metadiscourse into two categories, interactive and interactional, believing that “all metadiscourse refers to interactions between the writer and the reader” [29]. Interactive metadiscourse is oriented toward fostering text coherence to guide the reader in grasping the information presented, including transitions, frame markers and evidentials; and the interactional metadiscourse emphasizes the significance of reader participation, actualizing the metadiscoursal roles associated with stance-taking, involvement and evaluation, which are further classified into self-mentions, engagement markers, hedges, boosters and attitude markers. This classification not only emphasizes the interpersonal essence of metadiscourse but also specifies its formal reflexivity, clearly mirroring the writer’s keen awareness of the reader and modes of interaction. Interactional metadiscursive resources empower the writer to take part in a dynamic process of evaluation and engagement, foster solidarity, anticipate potential objections, and respond to an imagined dialogue with the reader [30]. The subcategory of interactional metadiscourse extends the scope of interpersonal metadiscourse research and highlights the social functions of metadiscourse by illuminating the interaction between the writer and the reader, which is adopted in this study.

In recent years, metadiscourse has been adopted as an analytical framework in studies across various contexts, including the academic, professional, commercial and media spheres [31–34]. In the business context, Incelli [35] conducts an investigation into the utilization of interpersonal markers within English and Italian tourism texts from three travel agencies, delving into the interactional metadiscourse strategies employed in tourist promotion; Huang et al. [36] further examines the occurrences and functions of interactional metadiscourse within travel blogs, exploring how they influence readers’ future travel experience. However, research into interactional metadiscourse in service discourse remains underdeveloped.

From the perspective of pragmatics, metadiscourse is the linguistic means used to realize metapragmatic purposes. Norton [37] notes that communicators not only exchange information through discourse but also construct their sense of identity. Metadiscourse conveys pragmatic information in the process of communication, expresses the speaker’s intention, and helps him to complete the communicative task smoothly [38]. Hyland [31] proposes that metadiscourse falls within the realm of pragmatics, maintaining that metadiscourse is the means used by the writer to express himself in the text, which needs to be examined in the context. From the perspective of social constructivism, the communicator’s identity is not preset or fixed but emerges in the process of communication [39], and it can be constructed by various metadiscourse resources [40]. Chen [41] expands the concept of metadiscourse, pointing out that communicators can use metadiscourse to achieve communicative goals such as discourse management and interactive evaluation, thus explicitly presenting or implicitly conveying pragmatic identity, that is, the identity that communicators dynamically choose and construct based on communicative needs [20].

Some scholars have begun to pay attention to the relationship between metadiscourse and identity construction, such as exploring the use of metadiscourse by advertisers to construct different identities for the purpose of persuading customers [42]; studying the key role of metadiscourse markers in constructing identities in research articles published on international journals [40]; elaborating on the utilization of metadiscourse resources by English as a foreign language instructor to construct identities of both a competent graduate student and a knowledgeable teacher [43]; exploring how e-commerce shopkeepers use metadiscourse to highlight self-identity in multiple dimensions in product descriptions [44]; and investigating how individuals discursively construct their identities on TikTok by enacting stance taking through both textual and nonlinguistic metadicourse [45].

Most of the previous studies have focused on qualitative analysis, exploring the use of metadiscourse to construct identity in different contexts to achieve the communicative goals. The appropriate use of interactional metadiscourse can promote the writer’s stance and solidarity with readers, thus persuading readers through shared attitudes and values and involving them collaboratively in textual constructions [46]. Despite the importance of interactional metadiscourse in identity construction and interpersonal communication, there remains a paucity of comparative research on the interactional metadiscourse used in business responses with different cultural backgrounds combining qualitative and quantitative methods. In the process of responding to online negative reviews, hotel responders not only communicate and interact with guests but also focus on restoring the image of the hotel and enhancing customers’ purchasing intentions [2]. Driven by these communicative needs, responders are justified in employing interactional metadiscourse dynamically to construct identities, referring to the dynamic pragmatic identity constructed under the drive of communicative needs [20].

Therefore, we aim to conduct a comparative study on Chinese and British hotel responders’ use of interactional metadiscourse and their constructed identities, employing both qualitative and quantitative methods to investigate their dynamic diversity and functions. By examining the similarities and differences in metadiscourse use and identity construction in responses to negative reviews between Chinese and British hotels, we can uncover the linguistic and communicative strategies that are unique to different cultural backgrounds [14]. This study not only advances current research on metadiscourse and identity in service contexts but also fosters understanding and cooperation among hotel industries across different cultural backgrounds, thereby enhancing our grasp of intercultural communication between the East and the West.

## 3. Research design

### 3.1 Data collection

The corpora of this study are selected on Tripadvisior.com, a leading travel website in the world, which mainly provides comments and suggestions on hotels, scenic spots, restaurants and other businesses from the perspective of consumers. The website has many hotel reviews and provides five hotel evaluation grades—“excellent”, “very good”, “average”, “poor” and “terrible”—for guests to choose, which has become an important source of information for consumers to choose hotels. In accordance with the ethical guidelines [47], the content on a web platform that is easily viewable to individuals without requiring membership or an account is not considered a closed or private group. On the website of Tripadvisor.com, the hotel responders engage with their guests through publicly accessible posts. Both the reviews and responses are intended for public viewing, implying that all the information can be utilized for research purposes without requiring informed consent [48].

To enhance the representativeness of our corpora, in June 2023, we selected the top ten five-star hotels (based on the ranking of the best value hotels on www.tripadvisor.com) respectively in Beijing in China and London in the UK, two first-tier cities in the world, for review analysis. Considering the more advanced state of the online business landscape in these metropolises, hotels ranking in the top ten of the five-star hotels represent high standards within the industry, tend to embrace a cutting-edge business philosophy and attract guests all over the world, thereby representing a broad and diverse customer base. Studying the hotel responses to negative reviews can provide insights into how responders uphold the hotel images and nurture harmonious relationship with guests through effective communication. To ensure the equivalence of corpus in data comparison, we only focused on the negative reviews and the corresponding responses written in English on the review pages of ten hotels in Beijing and London respectively, underscoring the global reach of the clientele and the responders’ keen awareness of addressing a vast, international audience through the universal language of English. While the shared language facilitates our quantitative analysis, our research focused primarily on identifying both similarities and differences in the metapragmatic awareness and stylistic preferences demonstrated by responders from varied cultural backgrounds when responding to negative reviews.

We first identified and extracted online reviews labeled with “poor” and “terrible” ratings by guests for hotels and then manually selected the hotels’ responses to the negative reviews, eliminating any duplicate response. Ultimately, we collected the first ten responses to negative reviews for each of the ten hotels across the two cities as corpus samples, and two corpora were established, namely, CORB (Corpus of responses to negative reviews from hotels in Beijing) and CORL (Corpus of responses to negative reviews from hotels in London). Each corpus consisted of 100 responses to negative reviews, and the time span of the responses was relatively large, covering from March 2021 to June 2024. The guests who left negative reviews included both domestic and international guests, representing a relatively broad customer base. The total word count for CORB amounted to 13,329, while the number of words in CORL reached 16,195. In order not to bring harm to the hotels, we decided to anonymize the hotels’ names to protect their privacy, and the hotel name was replaced by the letter X in the following corpus analysis.

### 3.2 Research questions

This study aims to answer the following two questions:

1. What are the characteristics of the interactional metadiscourse used in responses to online negative reviews of hotels in Beijing and London, and what are the similarities and differences?
2. What are the characteristics of the identities constructed by interactional metadiscourse in responses to online negative reviews of hotels in Beijing and London, and what are the similarities and differences?
3. What do the characteristics of interactional metadiscourse use and identity construction reveal about the distinct communication strategies and cultural values in China and the UK?

### 3.3 Data analysis

The metadiscourse we focused on in this study was based on the five subcategories of interactional metadiscourse in the metadiscourse classification of Hyland [29], because in responses to negative hotel reviews, responders mainly used interactional metadiscourse to construct identity, maintained hotel image and built a harmonious relationship with guests; and the frequency of interactional metadiscourse in the corpora was far greater than that of interactive metadiscourse. Quantitative analysis and qualitative analysis were used in this study.

To ensure the reliability of annotation, 20% of responses in each corpus were first annotated by the two authors according to Hyland’s [29] framework of interactional metadiscourse. Then, based on Chen’s [49] definition of pragmatic identity and the metadiscursive framework of identity [20], we identified and counted the types of identities constructed through interactional metadiscourse at the sentence level. We used the calculation method of percent agreement, and the intercoder agreement rates were 86% (concerning the coding of interactional metadiscourse) and 84% (concerning the identification of identity), indicating a high level of inter-coder reliability. After that, two authors independently coded the use of metadiscourse and identity construction in the two corpora. The inconsistencies were discussed until we reached a consensus. Next, chi-square tests using SPSS 26 were conducted to discern the similarities and differences in the frequency of interactional metadiscourse use and the emergence of identities within the two distinct corpora (the original frequency is counted according to the actual number of times it appears in a sentence). Because the number of words in CORB was not equal to that in CORL, the frequency of interactional metadiscourse was calculated every ten thousand words, and the frequency of identity construction was calculated according to the proportion of the total number of identities in each corpus to ensure their comparability. Furthermore, we adopted a qualitative approach to perform descriptive and comparative analyses on the selected discourse samples, which enabled us to investigate the hotel responders’ application of interactional metadiscourse and identity construction in greater depth, shedding light on their pragmatic functions and underlying communicative intentions.

## 4. Results

### 4.1 Use of interactional metadiscourse in responses to negative online reviews of Chinese and British hotels

#### 4.1.1 Characteristics of interactional metadiscourse in CORB and CORL

Upon examining the corpora, we find that interactional metadiscourse primarily serves several key functions in responses to negative hotel reviews. These functions include providing feedback, facilitating interpersonal communication, demonstrating commitment to action and offering suggestions, which mirror the communicative purposes inherent in the deployment of interactional metadiscourse by responders, namely, to rehabilitate the hotel’s image and build a harmonious relationship with guests. Based on the metadiscourse classification proposed by Hyland [29], interactional metadiscourse is subdivided into self-mentions, engagement markers, boosters, hedges and attitude markers, and the analysis framework of interactional metadiscourse used in the corpora is constructed, as shown in Table 1.

**Table 1:** Categories of interactional metadiscourse in CORB and CORL.

Category	Function	Example
Self-mentions	Refer to hotel	we, our, us, I, my, me
Engagement markers	Interact with the guest	you, your, guest, guests, please…
Boosters	Emphasis on certainty commitment and action	always, absolutely, certainly, indeed, very …
Hedges	Indicate the possibility of speculation	may, might, would, possible, seem to…
Attitude markers	Express apologies, thanks, expectations, etc.	sorry, disappointed, grateful, look forward to, sincerely hope…

In responses to negative reviews, responders engage with guests through self-mentions and various addresses, expressing concerns and empathy. They offer reasonable explanations for the issues raised, and provide prompt feedback along with suggestions for enhancement measures aimed at alleviating guests’ dissatisfactions. The following section will analyze the use of interactional metadiscourse in the corpora through the analysis of specific examples. It is worth noting that in the following pairs of examples, the first will be from a Beijing Hotel and the second from a London hotel.

(1) **We** are working on plans to uplift **our** facilities as a part of **our** continuous efforts to meet **our** guests’ expectations, so **we** hope you will feel more inspired and energized during your future stays with **us**.
(2) **My** team and **I** always available to speak about any queries or suggestions **our guests** may have for **us**, and **I** am so sorry to read that you did not feel able to share the extent of your concerns with any of **us** during your stay.

This pair of examples reveals the use of self-mentions in hotel responses. In example (1), the responder from the Beijing hotel uses the first-person plural “we”, “our” and “us”, signifying the collective identity of the hotel’s management team. It conveys the relevant information that plans are being made and enhancements to facilities are underway, all aimed at fulfilling the guests’ anticipations. Furthermore, it expresses the hotel staff’s expectation for the guests’ return, reflecting a forward-looking stance. The first-person collective perspective has the effect of empathy, contributing to conscious or unconscious efforts to gain the confidence of their interlocutors [50], which can shorten the distance between the two sides of communication. Generally, group commitment is more authoritative and credible. In example (2), the responder from the London hotel adopts the first-person singular form, demonstrating a manager’s concern for the guest’s questions and suggestions. The responder extends an apology to convey empathy, engaging in empathetic communication that shapes the image of a sincere and responsible manager [14], so as to gain the trust of guests and readers. In both cases, the responders address the other side as “our guests”, expressing a sense of closeness by substituting the appellation and pulling the relationship between the two sides into a unified front, thus eliminating misunderstandings and building a harmonious relationship with the guests.

(3) If **you** have any problems during **your** stay, **please** contact with our Guest Relations Manager directly.
(4) **Should you** consider returning and I would be most grateful if **you** would be kind enough to make **your** next reservation.

These two examples present the use of engagement markers, which are devices designed to explicitly address readers, either to focus their attention or include them as active participants within discourse, including second-person pronouns and imperatives [29]. In example (3), the responder from the Beijing hotel uses the second-person pronoun “you” to refer to the guest, and starts a direct dialogue with the imperative form “please contact with…” to suggest that the guest can contact the Guest Relations Manager if he encounters any problems during his stay. Imperatives are typically used to perform the speech acts of commanding, requesting and advising [51]. In this context, the imperative sentence can be regarded as a positive politeness strategy, which guides the guest to respond and carry out the action arranged by the speaker [52], effectively enhancing the guest’s participation. In example (4), the responder from the London hotel uses two conditional sentences, “should you…” and “if you…”, to express the expectation that the guest will reserve the hotel again, fostering positive interpersonal interactions with humility and warmth as well as persuading the guest to consume again.

(5) Whilst I **absolutely** understand how this affects your experience of Beijing, I also **must** highlight that the event influenced the entire city, with 5-star properties being affected across the board.
(6) Please allow me to assure you that it was **most certainly** not our intention to appear discourteous or unwelcoming and I would **very much** like to be able to personally speak to you.

Through the use of boosters, the responders in the two examples emphasize the certainty of the content, aiming to gain the recognition of the other party while enhancing the credibility of the information [29]. In example (5), the responder from the Beijing hotel uses “absolutely” to express full sympathy towards the guest’s dissatisfaction and tries to obtain the guest’s understanding. Then, the use of “must” serves to highlight the underlying causes of guest complaints, signifying that public events have had an impact on the city’s five-star hotels. A forthright attitude is adopted to demonstrate that the statement is an incontrovertible fact. In example (6), the responder from the London hotel uses the superlative “most certainly” to indicate that the hotel staff has absolutely no intention of being discourteous towards the guest. Furthermore, the use of “very much” conveys a keen desire to engage in dialogue with the guest and to elucidate the hotel’s managerial responsibilities. The responder engages in the negotiation and resolution of customer complaints with a focus on caring for the guest’s emotional needs, thereby upholding the hotel’s positive image.

(7) We did some investigation and there **seems to be** a complex chain of events and communication which led to the delay. If you wish you **may** reach out to me by email.
(8) Our usual check-in time is from 2 pm, and our housekeeping team do their very best to have the rooms ready before 2pm but this **may** not be always **possible**.

The responders in these two examples employ hedges to indicate speculation and propose various possibilities [29], reflected as the negotiation awareness and candid attitude of responders. By marking statements as provisional, hedges seek to involve readers as participants in their ratification, conveying deference, modesty or respect for the guests’ views. In example (7), the responder from the Beijing hotel acknowledges that the guest’s complaint about delays is caused by a series of events and poor communication. The use of “seems to be” avoids making absolute assertions, demonstrating objectivity, while “may” is used to express the desire for further communication with the guest, suggesting the possibility of contacting via email and resolving the issue, which shows the intent to facilitate a smooth resolution. In example (8), the responder from the London hotel explains the guest’s dissatisfaction regarding the inability to check into their room prior to the usual time. Even though the housekeeping team endeavors to expedite the preparation of rooms to the utmost extent, it is a widely observed convention for check-in times to commence after 2 p.m. The use of “may” and “possible” indicates that the guest’s request for early check-in cannot always be provided. By frankly admitting to incidents and issues as well as clarifying hotel check-in rules, the responder mentions the efforts made by the staff, skillfully using defensive rhetoric to minimize the negative impact of the review [8], which allows the guest to feel the sincerity of the hotel, making it easier to obtain the understanding and forgiveness of the guest and other readers.

(9) **Thank** you again and **sincerely apologized** for any inconvenience during your stay, **looking forward to** welcome you back to X.
(10) I was **glad** to read that you enjoyed a relaxing drink in our **wonderful** X, however I was equally **disappointed** to hear your comments about your breakfast experience.

This pair of examples shows the uses of attitude markers, which reveal one’s attitude towards a proposition, encompassing the expression of surprise, agreement, significance, responsibility, disappointment, and so on [29]. They are indicative of the communicator’s emotional responses, evaluative judgments and affective stances [53]. By using both positive and negative attitude markers, responders not only express their own feelings and attitudes but also convey understanding towards the guests from an empathetic perspective. This creates an emotional resonance that facilitates the negotiation and settlement of disputes. In example (9), the responder from the Beijing hotel first expresses gratitude for the guest’s feedback, then uses a negative attitude marker “apologized” to make sincere apologizing, and employs a positive attitude marker “looking forward to” to express anticipation for the guest’s return. By utilizing affective attitude markers, the responder establishes a shared emotional space with the guest, demonstrating the hotel’s respect for them. The positive politeness strategies used by the responder help to build solidarities with the guest, claiming common ground and creating intimacy between both parties [54]. In example (10), the responder from the London hotel begins with the positive attitude markers “glad” and “wonderful” to acknowledge the guest’s approval of the hotel’s beverages and bar services, establishing a positive image of the hotel. Besides, the responder uses the negative attitude marker “disappointed” to show empathy with the guest, expressing understanding for the unpleasant experience the guest has encountered during breakfast, which helps to alleviate the guest’s negative emotions and ease interpersonal relationships.

#### 4.1.2 Similarities and differences of interactional metadiscourse use in CORB and CORL

After identifying the categories of metadiscourse used in CORB and CORL, we count the normalized frequencies (per 10,000 words) of five types of interactional metadiscourse and conduct chi-square tests, as shown in Table 2. The raw data relevant to the study are provided in the S1 Data.

**Table 2:** A comparison of the frequencies of interactional metadiscourse between CORB and CORL.

Interactional metadiscourse	Subcategory and examples	CORB	CORL	x^2^	p-value
Self-mentions	The first-person plural: we, our, us	522.17	351.34	35.024	.000
	The first-person singular: I, my, me	176.31	292.68	23.643	.000
	Total	698.48	644.02	2.329	.127
Engagement markers	The second person: you, your	551.43	566.22	0.213	.644
	Addressing the other side: guest, guests	48.02	54.96	0.478	.489
	Imperative: please…	46.52	37.05	1.195	.274
Boosters	always, very, never, certainly, absolutely…	100.53	153.75	11.158	.001
Hedges	may, might, possible, somewhat, seem to…	104.28	59.90	11.902	.001
Attitude markers	Negative marker: sorry, apologize, disappointed, regret…	100.53	117.94	1.334	.248
	Positive marker: thank, glad, hope, look forward to…	257.33	197.59	7.829	.005

Self-mentions are frequently used in both corpora, with respective totals of 698.48 (per 10,000 words) and 644.02 (per 10,000 words), a difference that is not significant (x^2^ = 2.329, p = .127). However, the use of the first-person plural self-reference in CORB significantly exceeds that in CORL (x^2^ = 35.024, p = .000). This suggests that the responders from Beijing hotels tend to speak more as a collective management team to build a sense of solidarity [46], thereby emphasizing the leadership’s rallying power and the hotel’s unity. They provide detailed explanations for customer complaints from various perspectives, aiming to establish the hotel’s reputation as the core of authority and credibility. In contrast, the use of the first-person singular self-reference in CORL is significantly greater than that in CORB (x^2^ = 23.643 p = .000), which indicates that the responders from London hotels focus more on demonstrating their individual capabilities and initiatives as managers. The social and psychological distance between responders and guests are narrowed, fostering positive interpersonal communication.

In terms of engagement markers, the utilization of second-person stands out as the most prevalent among both parties in their responses to negative reviews, with respective totals of 551.43 (per 10,000 words) in CORB and 566.22 (per 10,000 words) in CORL, which are similar in frequency (x^2^ = 0.213 p = .644). This indicates that responders from both sides place emphasis on communication with guests, enhancing their sense of involvement in the hotel matters through direct dialogues, addressing and resolving issues within an interactive context. In addition to the second-person reference, responders from both sides use singular and plural forms of guest with similar frequency (x^2^ = 0.478 p = .489), maintaining a formal tone to convey respect. The frequency of using imperative sentences to interact with guests by both sides is also similar (x^2^ = 1.195 p = .274). The responders employ imperative sentences to offer suggestions, provide contact methods or offer solutions, demonstrating an attempt to promptly address customer complaints and grievances, while simultaneously focusing on the restoration of the hotels’ images [55]. In both corpora, the frequency of the three subcategories of engagement markers shows no significant difference, suggesting that responders from both sides have adopted similar communicative approaches. These approaches involve guests in the discourse, ensuring smooth communication and ultimately repairing the relationship with guests.

The frequency of boosters in CORL is significantly greater than that in CORB (x^2^ = 11.158 p = .001), suggesting that London hotel responders are more inclined to express certainty in their statements. This serves to increase the credibility of hotel management by conveying information with conviction to persuade the guests of their problem-solving capabilities. In contrast, the frequency of hedges used in CORB noticeably exceeds that in CORL (x^2^ = 11.902 p = .001). Hedges typically function to soften the tone or indicate a conservative attitude when expressing propositions. While guiding and indicating the pragmatic intentions of the other party, the use of hedges alleviates the imposition of face-threatening acts [56], thus maintaining the relationship between the two parties [57]. The utilization of hedges helps to open a discursive space where guests can dispute the responders’ interpretations. Beijing hotel responders are adept at using vague adverbs and modal words to craft a humble image, guiding guests to feel empowered to question and challenge in a reasonable and orderly manner, thereby fostering trust in the hotel’s sincerity and genuine apology.

Regarding the use of attitude markers, responders from both sides employ positive attitude markers more frequently than negative markers, indicating a preference for addressing customer dissatisfaction with a proactive and solution-oriented approach. The frequency of negative attitude markers used in CORB and CORL is similar (x^2^ = 1.334 p = .248). Responders from both sides mainly express empathy and acknowledge the guests’ feelings of annoyance, disappointment or irritation, aiming to soothe and reassure the guests. However, the frequency of positive attitude markers in CORB exceeds that in CORL (x^2^ = 7.829 p = .005), exhibiting a significant difference. It is obvious that responders from Beijing hotels place greater emphasis on expressing gratitude, pleasure, hope and other positive emotions, to create a harmonious atmosphere and evoke favorable emotional responses from guests, hence increasing their approval in how the hotels handle issues.

### 4.2 Identities constructed by interactional metadiscourse in responses to negative online reviews of Chinese and British hotels

#### 4.2.1 Types and characteristics of identities constructed in CORB and CORL

In this study, we consider identity as the pragmatic identity, referring to the dynamic identity constructed in the interaction and chosen by the communicator to perform the corresponding communicative functions [49]. Driven by the communicative purposes, communicators can use metadiscourse to explicitly construct various identities [41]. There is no fixed linguistic code for what kind of metadiscourse constructs what kind of identity. However, based on Chen’s [20] prior framework for metadiscursive construction of identity, which involves differentiating identity categories by the communicative functions enacted by communicators [58], our detailed analysis of the two corpora reveals that the hotel responders employ interactional metadiscourse to fulfill four main communicative functions: delivering feedback, fostering interpersonal communication, committing to actions and making suggestions. Consequently, we identify four corresponding pragmatic identities: manager, communicator, doer and advisor. We read the corpora sentence by sentence to determine which identity the responder constructs, and the original frequency of identity is counted by their actual occurrences in a sentence. The analytical framework is shown in Table 3.

**Table 3:** Types of identities constructed in CORB and CORL.

Identity	Metadiscourse	Communicative function	Example
manager	Please…, our, sincerest, you	Admitting inconvenience, providing apology	On behalf of the entire team and management, please accept our sincerest apologies for the technical inconvenience that you encountered.
communicator	You, my, I, may, most convenient	Expressing the willingness to communicate	If you are agreeable to accepting my telephone call, may I ask that you kindly advise me of your number and the most convenient time for me to place the call.
doer	We, our, thorough, immediate, all	Launching investigation, promising action	We are conducting a thorough investigation to verify the comments and have taken immediate steps to arrange refresher training on room cleaning procedures for all stylist in our X hotels.
advisor	Especially, possible, us, your, might	Offering suggestions on arranging accommodation	It is recommended, especially at this time of year, to book ahead if at all possible or let us know during check-in what your plans might be.

It is evident that the responders from both Chinese and British hotels employ a comprehensive use of interactional metadiscourse to construct four identities so as to realize the communicative goals of maintaining reputation and building harmonious relationships with guests.

(11) **I** will address **your** concerns with our Front Desk staff to ensure that in **any** situation, **we** go above and beyond in assisting **our guest** in **any** way **possible**.
(12) Whilst **my** team and **I** remain **disappointed** not to have had the chance to learn earlier and address any concerns **you** had, **we of course** will take on board **your** feedback.

In both instances, the responders construct the identity of manager using self-mentions, engagement markers, attitude markers, hedges and boosters. Both parties explicitly construct the identity of manager with first-person pronouns, as well as the second-person pronoun “you” and possessive adjective “your”, indicating a willingness to listen to customer issues and provide feedback. This reflects highly interactive verbal characteristics that uphold guests’ positive face [57]. By integrating guests into the process of discussing and resolving issues, the responder from the Beijing hotel in example (11) uses hedges such as “any” and “possible” to perform the role of a responsible manager, suggesting an intent to assist the guest as much as possible. On the other hand, in example (12), the responder from the London hotel initially employs a negative attitude marker “disappointed” to express regret for not resolving the issue earlier, followed by the booster “of course” to emphasize the hotel’s regard for the guest’s opinions, thereby better meeting the guest’s emotional needs and leading to increased guest satisfaction and loyalty.

(13) Thank **you** very much for **your** support and understanding, and **I sincerely hope** to have the pleasure of talking to **you** soon.
(14) Thank **you** for **your** review. **I** am very **sorry** about what happened, so **please** allow **me** to try to resolve this matter for **you** personally.

By using engagement markers, self-mentions and attitude markers, the responders in the two examples thank the guests and invite them to talk, constructing the identity of communicator. Both responders express gratitude for the guests’ understanding, support and suggestions by adopting a grateful repair approach. The responders take the guests’ views into account, acknowledge the inconveniences brought to the guests, and demonstrate empathy and appreciation for their reviews, all aimed at compensating for any losses incurred and achieving interpersonal balances [59]. In example (13), the responder from the Beijing hotel uses “sincerely hope” to indicate a desire to further discuss the issue with the guest, creating a sense of intimacy and thus appealing to their shared emotional bonds. In example (14), the responder from the London hotel uses “sorry” to express regret for the dissatisfaction caused to the guest, and uses an imperative sentence to express a willingness to personally solve the problem for the guest. Both parties focus on using interactional metadiscourse to establish a closer relationship with guests, demonstrating an honest and sincere communicator identity. By engaging in conversation that is empathetic, respectful and inclusive, they create a space where guests feel valued, heard and understood.

(15) **We** will **most certainly** take this as a learning opportunity for the team and evaluate **our** shortcomings to **continuously** improve the standards of **our** products and offerings.
(16) **We** are **constantly** investing in all areas of the hotel and have a comprehensive programme to ensure that all our suites are kept at the **highest** standard to meet **our** guests’ expectations.

In these two instances, both responders use the first-person plural pronoun to refer to the management teams, including themselves, employing the boosters of “most certainly” and “highest” to pledge that the hotels will definitely treat customer dissatisfaction and complaints as learning opportunities to inspect any shortcomings, and ensure that the rooms will meet the highest standards. The use of boosters not only underscores the responders’ confirmation of their truthful statements, but also reflects their consideration of subsequent actions and hotel reputation [60], thereby constructing the identity of doer. According to Martin and White [61], the concept of attitude moves beyond emotion to deal more comprehensively with feelings, including affect, judgement and appreciation; expressions of usuality and inclination can be related to judgements of normality and tenacity. In order to comfort the guests, the hotel responders use “continuously” and “constantly” to promise that they will continually try their best to improve the hotel’s facilities and services. The attitudinal adverbs are used to express the responders’ positive evaluations and good intentions for the benefit of guests, contributing to good relationships between the two sides [32]. It is obvious that both parties emphasize decisive and robust actions, using self-mentions, attitude markers and boosters to construct an efficient doer identity.

(17) **Please** feel free to contact **us** if **we may** be of any further assistance.
(18) **Should you** require any additional assistance, **please** let **us** know by contacting **us** directly at the hotel.

In both cases, the responders use engagement markers, self-mentions and hedges to construct the identity of advisor. They demonstrate a strategic use of language that facilitates a constructive dialogue with the guests. As “please” is often used when constituting requests, the imperatives are expressions of friendly advices [51]. Here, by employing imperatives such as “please…”, the responders signal a polite and respectful request for engagement, indicating a willingness to attend to the guests’ concerns and invite the guests to contact the hotels. As responders aim to provide guests with promises about their efficient service and maintain close relationships with potential guests, they employ the solidarity politeness strategy, such as the use of inclusive pronouns [62]. The use of self-mention, such as “we” or “us”, represents the responders’ request for contact with the guests on behalf of the hotels and serves to promote the interaction by projecting a unified front. Additionally, both parties use hedges such as “may” and “should” to soften the tone to express respect and politeness, mitigate the threat to the guests’ face and maintain interpersonal harmony in the current context, making it easier for the other party to accept rather than press the guests.

#### 4.2.2 Comparative analysis of the frequencies of identities constructed in CORB and CORL

Upon meticulous observation and analysis of the two corpora, we enumerate the frequencies of occurrences for four types of identities—manager, communicator, doer and advisor—constructed through interactional metadiscourse in CORB and CORL, as shown in Table 4. It is clear that the frequencies of identities in responses to negative reviews not only share some commonalities, but also exhibit certain differences between Chinese and British hotels.

**Table 4:** A comparison of identity occurrences between CORB and CORL.

Identities	CORB	CORL	x^2^	p-value
manager	198 (29.46%)	101 (12.74%)	7.715	.005
communicator	278 (41.37%)	346 (43.63%)	0.184	.068
doer	127 (18.90%)	255 (32.16%)	4.448	.035
advisor	69 (10.27%)	91 (11.48%)	0.053	.818

In terms of proportion, the identity of communicator is most frequently constructed through interactional metadiscourse in both corpora, with little variance in frequency (x^2^ = 0.184, p = .668). This suggests that responders from both sides place a significant emphasis on their interaction with guests. Within specific contexts, the identities constructed by participants in communication can become a communicative resource or strategy for cultivating and sustaining interpersonal relationships. As communicators, both parties use apologies and expressions of gratitude to demonstrate concern for guests’ emotions and feelings. This shows the hotels’ willingness to admit their shortcomings and publicly apologize with sincerity, thus bridging the gap with guests so as to establish emotional connections. The proportion of manager identity constructed by Beijing hotel responders is significantly greater than that of London hotel responders (x^2^ = 7.715, p = .005), indicating that they are more inclined to act on behalf of the hotels in offering feedback and responses. This reflects their serious attitude toward addressing guest concerns and issues, aiming to save face for themselves, striving to defend the hotels’ reputation, and rebuilding guests’ confidence in the hotels and their services [5]. The proportion of doer identity constructed by London hotel responders is greater than that constructed by Beijing hotel responders, with a noticeable difference (x^2^ = 4.448, p = .035). This reflects that London hotel responders focus more on committing to follow-up actions, efficiently handling customer complaints and grievances, and providing acceptable and effective problem solutions. The proportion of advisor identity constructed by both Beijing and London hotel responders is the lowest, with frequencies being close (x^2^ = 0.053, p = .818), indicating that the purpose of the responders’ timely suggestions is not to pressure guests, but to prioritize their interests [63], upholding their right to be informed fairly and to autonomously solve the problems.

## 5. Discussion

The above analysis of interactional metadiscourse use and identity construction in responses to negative online reviews from Beijing and London hotels reveals intriguing strategies and cultural differences in how hotels manage their online reputation and customer relationships. The categories of interactional metadiscourse identified in this study—self-mentions, engagement markers, boosters, hedges and attitude markers—are instrumental in shaping the hotel responders’ identities. Most self-mentions help to establish connections with guests, emphasizing the hotels’ commitment to addressing issues and improving services. Engagement markers, such as the second-person pronouns and imperatives, directly involve guests in the conversation, fostering a sense of inclusivity and encouraging active participation. Boosters and hedges are used tactically to either emphasize certainty and conviction or cautiously manage expectations, respectively. Attitude markers reveal the hotel responders’ negative and positive emotions, allowing for the expression of empathy, concern, gratitude and apology, which are vital for attempting trust repair [64] and maintaining positive images of the hotels.

By comparing the use of the interactional metadiscourse in the two corpora, it is revealed that both Beijing and London hotel responders employ similar strategies in terms of engagement markers, using second-person pronouns to involve guests directly in the conversation. This suggests that regardless of the cultural background, inclusivity and direct dialogue are universally valued in hospitality communication. Beijing hotel responders’ preference for first-person plural form and indirect expression indicates a collective approach to addressing issues, which may be rooted in Chinese cultural values that emphasize group harmony and face-saving [65]. It aligns with the concept of Chinese rapport, which places importance on interpersonal relationships and the maintenance of images [46]. In contrast, London hotel responders’ use of first-person singular pronouns and explicit expressions reflects a more individualistic approach, which is consistent with Western cultures that value personal accountability and directness.

The use of boosters by London hotel responders demonstrates a tendency to express certainty and conviction, which may be perceived as assertiveness in Western cultures but is interpreted as overconfidence in Eastern cultures. On the other hand, the greater use of hedges by Beijing hotel responders reflects a more cautious and conservative approach, which is in line with the cultural values of avoiding confrontation and maintaining politeness. These differences indicate that although the functions of interactional metadiscourse are universally applicable, the forms and frequencies of metadiscourse differ across cultures. The more frequent use of positive attitude markers by Beijing hotel responders signifies an intention to foster a harmonious atmosphere and build trust through positive emotional connections, which may be particularly effective in cultures where maintaining harmony is highly valued. London hotel responders also use positive attitude markers but to a lesser extent, suggesting a more balanced approach to emotional expression. The cultural sensitivity is important in the service industry’s communication practices, which reminds the hotel responders to be sensitive to their guests’ expectations, employing the proper interactional metadiscourse to effectively address negative feedback and foster customer loyalty.

For identity construction, the hotel responders from both sides actively construct four types of identities—manager, communicator, doer and advisor—through the strategic use of interactional metadiscourse. These identities perform the pragmatic functions of providing feedback, engaging in interpersonal communication, committing to actions and offering suggestions. The hotel responders employ interactional metadiscoure to construct the identity of manager with the frequent use of first-person pronouns and possessive adjectives with an inclusive atmosphere, reflecting a high level of interactivity and a commitment to addressing guest concerns. The use of hedges suggests a willingness to open the dialogic space [66], which is crucial for building trust and credibility. By using engagement markers and attitude markers, the responders express gratitude and a willingness to communicate, thereby constructing the identity of communicator. They not only acknowledge the guests’ complaints but also aim to compensate for any inconveniences, demonstrating empathy and appreciation. In the service industry, deploying attitude metadiscourse that resonates with shared emotional bonds proves particularly effective, given that customer satisfaction is profoundly connected to emotional experiences [30]. Moreover, the hotel responders emphasize decisive actions by using self-mentions, attitude markers and boosters to construct the efficient doer identity. This shows a commitment to ongoing efforts to resolve issues and improve services, which is essential for maintaining a positive reputation and fostering a sense of dedication to guests. In addition, the hotel responders construct the advisor identity through the use of engagement markers, self-mentions and hedges. They not only offer assistance but also seek to maintain close relationships with potential guests, demonstrating the importance of solidarity politeness strategies. It is worth mentioning that imperatives are used to offer polite advices, indicating respectful requests for engagement.

By comparing the identities constructed in responses to negative reviews from both Chinese and British hotels, it is evident that the cultural context influences the frequency and construction of identities. The manager identity is crucial for demonstrating accountability and commitment to addressing guest feedback. London hotel responders use direct expressions in the process of constructing manager identity, whereas Beijing hotel responders convey similar messages with an alleviating tone, considering the cultural emphasis on relational management [67]. Responders from both sides aim to establish a communicative bond with guests through the construction of the communicator identity. Nonetheless, London hotel responders often feature overt expressions of appreciation or regret, aligning with a more individualistic approach to customer service. On the other hand, Chinese responders may prioritize relational aspects, utilizing the interactional metadiscourse that emphasizes indirect expressions and collective stances. This finding is in line with the research of Hofstede et al. [68], which indicates that the individualism index for Mainland China is 20, suggesting a strong collectivist tendency; in contrast, the individualism index is 89 in the UK, reflecting a robust individualistic orientation.

Action orientation is also a key aspect in constructing the doer identity in both Chinese and British cultures. However, the way actions are presented reflects cultural differences. London hotel responders use direct imperatives or declarative statements to showcase actions, while Beijing hotel responders might employ more indirect means, suggesting actions through context rather than explicit commands. For the construction of the advisor identity, offering advices is a common strategy, but the manner of doing so reflects different cultural etiquettes. Beijing hotel responders frame advices with politeness strategies that prioritize deference to the guests’ perspective, while London hotel responders often offer direct recommendations. The multifaceted identity construction in responses to negative hotel reviews is a significant finding, as it demonstrates the complexity of maintaining positive hotel images and harmonious customer relationships within the digital realm. The findings also reveal that the hotel responders to negative reviews from different cultural backgrounds tend to use varied linguistic styles, such as formal or cordial tone, direct or euphemistic expression, and their linguistic choices are often closely related to the particular cultural values and social norms. Understanding the pragmatic tendencies and preferences of communicators in different cultural contexts can help enhance people’s sensitivity and adaptability in cross-cultural environments, thereby facilitating effective communications.

## 6. Conclusion

This study uses the corpora of responses to online negative reviews from hotels in Beijing and London, focusing on the use of interactional metadiscourse and identity construction in the discourse of responders. The findings collaborate with previous explorations into the interconnection between metadiscourse use and identity construction [40, 43, 69], thereby reinforcing that language used reflexively is a crucial vehicle for communicators to convey and realize the communicative purposes.

Combining discursive analysis and statistical data, we analyze the categories and characteristics of interactional metadiscourse and identities constructed by both Beijing and London hotel responders, as well as the similarities and differences between the two parties in a pragmatic sense, which contribute to an enhanced comprehension of the responders’ metapragmatic consciousnesses in constructing identities through interpersonal interactions [20]. The use of interactional metadiscourse and identity construction among Chinese and British hotel responders is a dynamic process of selection. Both sides focus on communicating and interacting with guests, openly expressing their feelings to alleviate guests’ negative emotions, opportunely making suggestions to maintain guest trust [70] and attract new guests [71]. Beijing hotel responders place more emphases on objective and detailed explanations for addressing customer complaints in the collective role of management, with a humble tone to avoid absolute conclusions and a positive attitude to shape the hotels’ positive images. In contrast, London hotel responders highlight the individual organizational and problem-solving abilities of managers to quickly respond and take action to gain guests’ understanding and recognition. These findings reveal that effective uses of discourse based on a metapragmatic awareness in responses to negative reviews prompt the improvement of the service quality as well as customer satisfaction and loyalty. Positive responses to negative reviews can motivate the hotels to engage in healthy competition, helping to promote the sustainable development of the service industry.

Despite the endeavors and claims that we have made thus far, the present study is limited in three ways. Initially, the corpora are confined to hotel responses to negative online reviews of a single Chinese and British city, potentially restricting the diversity of interactional metadiscourse usage and identity construction due to the limited scope of the data. Second, the current analysis does not incorporate the guests’ negative reviews presented on the webpage, which may play a supportive role in the responders’ use of interactional metadiscourse and identity construction, warranting further investigation. Finally, in alignment with the communicative objectives of enhancing hotel reputation and building rapport with guests, our focus has been exclusively on the use of interactional metadiscourse that fosters positive identity construction and interpersonal harmony, without considering whether there are any instances of interactional metadiscourse that may construct negative identities or adversely impact the relationship with guests. Despite its limitations, this study further clarifies the relationship between interactional metadiscourse and identity construction in responses to negative hotel reviews, enriching the research on commercial institutions’ metadiscourse use and identity construction from a pragmatic perspective. It can provide a reference for communicators to use metadiscourse to shape positive images and maintain harmonious relationships with customers. Future research could compare the similarities and differences in the use of metadiscourse and identity construction by commercial institutions in different contexts, and consider a broader range of cultural contexts to further explore the influence of culture on interactional metadiscourse or explore the correlation between the metadiscourse use and the communicative context, thereby offering insights for the pragmatic study of business discourse.

## Supporting information

S1 Data (XLSX)
